# Imaging molecular geometry with electron momentum spectroscopy

**DOI:** 10.1038/srep39351

**Published:** 2016-12-22

**Authors:** Enliang Wang, Xu Shan, Qiguo Tian, Jing Yang, Maomao Gong, Yaguo Tang, Shanshan Niu, Xiangjun Chen

**Affiliations:** 1 Hefei National Laboratory for Physical Sciences at Microscale and Department of Modern Physics, University of Science and Technology of China, Hefei, Anhui, 230026, China; 2 Synergetic Innovation Center of Quantum Information and Quantum Physics, University of Science and Technology of China, Hefei, Anhui 230026, China

## Abstract

Electron momentum spectroscopy is a unique tool for imaging orbital-specific electron density of molecule in momentum space. However, the molecular geometry information is usually veiled due to the single-centered character of momentum space wavefunction of molecular orbital (MO). Here we demonstrate the retrieval of interatomic distances from the multicenter interference effect revealed in the ratios of electron momentum profiles between two MOs with symmetric and anti-symmetric characters. A very sensitive dependence of the oscillation period on interatomic distance is observed, which is used to determine F-F distance in CF_4_ and O-O distance in CO_2_ with sub-Ångström precision. Thus, using one spectrometer, and in one measurement, the electron density distributions of MOs and the molecular geometry information can be obtained simultaneously. Our approach provides a new robust tool for imaging molecules with high precision and has potential to apply to ultrafast imaging of molecular dynamics if combined with ultrashort electron pulses in the future.

The physical and chemical properties of molecules directly depend on their geometries and electronic structures that both have always been the central issues in molecular physics. The geometry of a molecule is conventionally obtained by the methods of X-ray^1^,^2^ or electron diffraction^3^,^4^,^5^,^6^, from which the atomic positions are determined with sub-Ångström spatial resolution. An alternative imaging approach emerged in the past decade, which is referred to as the laser induced electron diffraction^7^,^8^,^9^,^10^,^11^, has also been demonstrated to image molecular structures with sub-Ångström precision. In this technique, an intense laser field is employed to extract electron from a molecule itself, and within one laser period a fraction of the tunneled electron wave packet will be forced back to re-collide and diffract from the parent molecular ion. The well-established method in the conventional electron diffraction is then applicable to retrieve the bond lengths of molecule.

On the other hand, the tunneled electron wave packet that directly emerges into the vacuum retains information about the orbital from which the electron is ionized^9^. By measuring the momentum distribution for these direct electrons, the fingerprint of the highest occupied molecular orbital can be observed through the filter of the suppressed binding potential through which the electron tunnels^9^. Thus one set of measurements simultaneously identifies the orbital wavefunction of molecule and the position of the atoms in the molecule in this laser induced electron tunneling and diffraction technique. Information about the ionizing orbital of neutral molecule is also imprinted on the high-harmonic radiation produced by the recombination of the re-collision electron with the parent ion in the laser field and allows the three-dimensional shape of the highest electronic orbital to be measured^12^.

Electron momentum spectroscopy (EMS), which is based on the electron-impact single ionization or (*e*, 2*e*) experiment near the Bethe ridge, is a well-established technique that can obtain the spherically averaged electron momentum distributions, or electron momentum profiles (see Supplementary Information Note 1), for any individual molecular orbitals (MOs) in principle^13^,^14^,^15^. This unique ability of imaging MOs makes the EMS a robust technique for exploring the electronic structures of molecules in gas phase^16^. However, the geometry information of molecule is usually veiled due to the single-centered character of the momentum space wavefunction for MO. In momentum space, for a MO which can be approximated by a linear combination of atomic orbitals (LCAOs), the information about the equilibrium nuclear positions ***R***_*J*_ is only present in the phase factors exp(−*i**p*** ⋅ ***R***_*J*_) introduced by Fourier transform of the wavefunction from position space to momentum space (see Methods for details). Therefore the electron momentum distribution of a MO will be modulated by a cosine or sine function with periodicity of 2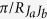, where 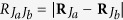 is the distance between atoms *J*_*a*_ and *J*_*b*_. This oscillation phenomenon is usually referred to as bond oscillation^17^, which can also be regarded as a result of the Cohen-Fano type^18^ or the Young-type interference effect originated from the coherent superposition of the (*e*, 2*e*) amplitudes from the atoms in the molecule. This type of molecular scale interference was first proposed by Cohen and Fano^18^ in photoionization and was successively demonstrated in the ionization of molecules induced by heavy ions^19^,^20^,^21^,^22^,^23^,^24^,^25^, photons^26^,^27^,^28^,^29^,^30^,^31^,^32^,^33^,^34^,^35^, as well as electrons^36^,^37^,^38^.

In the EMS experiments, the interference effect was first discussed in the 1980 s^17^ and clearly observed only recently in the experiments of CF_4_^37^, H_2_^38^,^39^, and SF_6_^40^,^41^. Direct observation of the interference pattern in electron momentum distribution is usually very difficult due to the weak modulation on the rapidly decreasing intensity at large momentum. The feasible way is to compare the experimental cross section of a molecule with the theoretical or experimental one-center atomic cross section^37^,^39^,^40^ or to compare the cross sections between two different vibrational states^38^. Kushawaha *et al*.^33^ in their photoionization work suggested a more obvious way to observe the interferences by measuring the ratio of two cross sections corresponding to the MOs with symmetrical and anti-symmetrical characters, which are expected to give oscillations in antiphase, thus magnifying the interference pattern.

In the present work, the similar scheme has effectively been applied in EMS experiments to uncover the multi-center interferences in CF_4_ and CO_2_. The scheme is pictorially illustrated in Fig. 1a. With CF_4_ as an example, the three outermost MOs (1t_1_, 4t_2_, 1e) of this molecule are essentially due to lone-pair electrons or 2*p* atomic orbitals (AOs) on the F atoms. Figure 1b shows the calculated electron momentum profiles (see Supplementary Information Note 2) for 4t_2_ and 1e orbitals at equilibrium geometry. In the logarithmic coordinate both of the momentum profiles show weak oscillations extending to large momentum region due to the multi-center interferences from the ionization of the four F atoms. Different orientations of the constituent 2*p* AOs in 4t_2_ and 1e orbitals lead to the oscillations almost completely in antiphase (Fig. 1b)^37^. The interference pattern can be significantly magnified by plotting the ratio of the momentum profiles for these two MOs, as illustrated in Fig. 1a and Fig. 1c. A very sensitive dependence of the oscillation interference pattern on the interatomic F-F distance is expected, as shown in Fig. 1c by the clear shift in fringe maximum by about 0.15 a.u. for small changes of 0.1 Å in *R*_FF_. Therefore, this dependence may provide a means of accurate determination of molecular geometries with sub-Ångström precision.

In this study, the accurate measurements are carried out for CF_4_ and CO_2_ by using a high-sensitivity angle and energy dispersive multichannel electron momentum spectrometer with simultaneous detection in 2*π* angle range^42^. Two-dimensional (2D) electron density map of binding energy and relative azimuthal angle for the outer-valence MOs for these two molecules have been obtained. The experimental electron momentum profiles for the relevant MOs are extracted. A strong dependence of the oscillation period on the interatomic distance is observed in the ratios of electron momentum profiles between two MOs with oscillations in antiphase, which is used to determine F-F distance in CF_4_ and O-O distance in CO_2_ with sub-Ångström precision. Thus, in our new approach, we can simultaneously obtain the electron density distributions of MOs and the molecular geometry information in one set of measurements. Benefited from the wide momentum range (from 0 to 8 a.u.) of this new version EMS spectrometer^42^, more than two periods of oscillations are included in the interference fringes. Besides, the present observation of interference effect totally depends on the experimental measurements and does not rely on the comparison with the one-center atomic cross section. These features make our approach a robust tool for imaging molecules with high precision and has the potential to apply to ultrafast imaging of molecular dynamics if combined with the ultrashort electron pulses^43^ in the future.

## Results

### 2D electron density maps

Figure 2a and b show the 2D electron density maps for CF_4_ and CO_2_ measured at impact energy of 1.2 keV plus binding energy (see Methods). These 2D maps are the (*e*, 2*e*) TDCSs as functions of binding energy and relative azimuthal angle *ϕ* (i.e. the momentum of the orbital electron) and contain all the information on binding energy spectra (BES), electron momentum distributions, and symmetries for various ionization states. Figure 2c and d show the total BES summed over all the measured *ϕ* for CF_4_ and CO_2_ respectively. Gaussian functions as shown by the solid curves, which correspond to the ionizations from different MOs, are invoked to fit the BES. The MO specific electron momentum profiles can be extracted by deconvoluting the corresponding ionization peaks of the BES at different *ϕ* and plotting the area under the fitted peaks as a function of the magnitude of momentum ***p*** (see Supplementary Information Note 2).

### Multicenter interference effect

**Figure 1:**
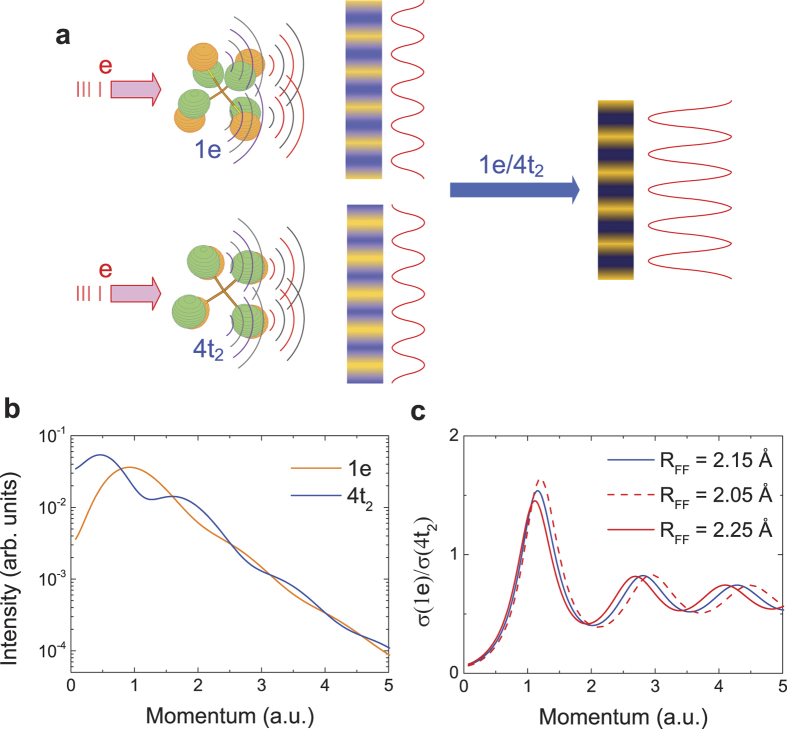
Multi-center interference in electron impact ionization of molecule. The CF_4_ molecule is adopted as an example. (**a**) Schematic representation of the multi-center interference and magnification of the interference pattern. (**b**) Logarithmically scaled electron momentum distributions for 1e and 4t_2_ MOs of CF_4_. (**c**) Ratios of momentum profiles at differential interatomic distances of CF_4_.

**Figure 2:**
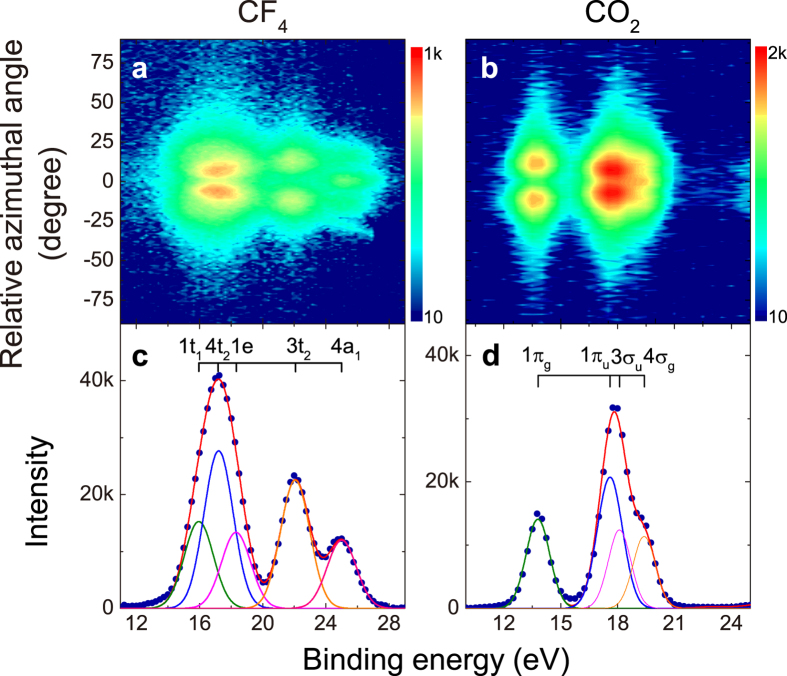
Two-dimensional electron density maps and total binding energy spectra. The two-dimensional electron density maps as functions of binding energy and relative azimuthal angle for (**a**) CF_4_ and for (**b**) CO_2_. The total binding energy spectra summed over all the measured azimuthal angle for (**c**) CF_4_ and for (**d**) CO_2_.

**Figure 3:**
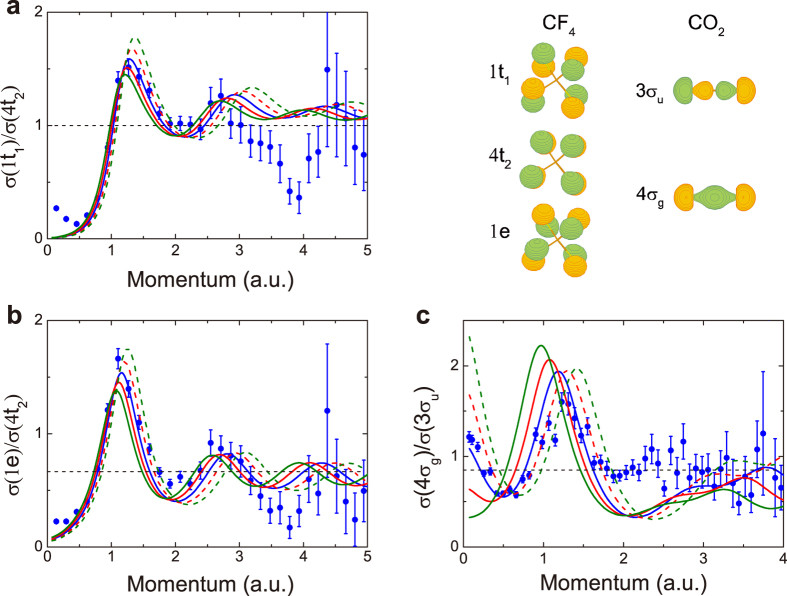
Experimental momentum profile ratios (solid circles) in comparison with theoretical ones (solid and dashed lines) at different interatomic distances. (**a**) The ratio of MOs 1t_1_ to 4t_2_ and (**b**) the ratio of MOs 1e to 4t_2_ of CF_4_. (**c**) The ratio of MOs 4*σ*_*g*_ to 3*σ*_*u*_ of CO_2_. The solid blue lines show the theoretical momentum profile ratios of the molecules at equilibrium geometries (*R*_FF_ = 2.1551 Å^44^ for CF_4_ and *R*_OO_ = 2.3267 Å^44^ for CO_2_). The solid red and green lines represent the theoretical results at *R*_FF_ = 2.1551 Å + 0.1 Å, *R*_FF_ = 2.1551 Å + 0.2 Å for CF_4_ and *R*_OO_ = 2.3267 Å + 0.1 Å, *R*_OO_ = 2.3267 Å + 0.2 Å for CO_2_. The dashed red and green lines show the theoretical results at *R*_FF_ = 2.1551 Å − 0.1 Å, *R*_FF_ = 2.1551 Å − 0.2 Å for CF_4_ and *R*_OO_ = 2.3267 Å − 0.1 Å, *R*_OO_ = 2.3267 Å − 0.2 Å for CO_2_. The inset locates at the right top of the figure show the orbital images of the involved MOs of CF_4_ and CO_2_.

The orbital images for 1t_1_, 4t_2_, 1e MOs of CF_4_ and for 3*σ*_*u*_, 4*σ*_*g*_ MOs of CO_2_ are shown at the top right of Fig. 3. For CF_4_ molecule, the three outer most MOs, 1t_1_, 4t_2_ and 1e, are composed of 2*p* lone-pair electrons on the F atoms. As we have mentioned, both the momentum profiles for 4t_2_ and 1e orbitals show weak oscillations due to the multi-center interferences from the ionization of the four F atoms. The phase of the interference factor depends on the different orientations of the constituent 2*p* AOs in the MOs^37^. In 4t_2_ orbital the 2*p* AOs of the four F atoms orient parallel to each other, while in 1e orbital the 2*p* AOs of each two pairs of F atoms are in opposite orientations. The different orientations lead to the interference oscillations of momentum profiles almost completely in antiphase (Fig. 1b). Besides 4t_2_, 1e orbital pair, the momentum profiles of 1t_1_, 4t_2_ orbital pair of CF_4_ and 3*σ*_*u*_, 4*σ*_*g*_ orbital pair of CO_2_ are also modulated by the interference factors in antiphase (see Supplementary Information Note 3 and Fig. S1 for detail).

The interference pattern will significantly be magnified by plotting the ratio of the momentum profiles as indicated in Fig. 1c. Figure 3a and b show the ratios of the measured momentum profiles *σ*(1t_1_)/*σ*(4t_2_) and *σ*(1e)/*σ*(4t_2_) for CF_4_ by solid circles. Both ratios exhibit significant oscillations around constant values with more than two periods, which is the distinct evidence of the multi-center interference effect. The constant is the product of the ratio of the electron occupation numbers of MOs (6 for 1t_1_, 4t_2_ and 4 for 1e) and the ratio of the pole strengths of the corresponding ionization peaks. The pole strengths of the main ionizations peaks for the outer valence orbitals of molecules are usually approximately equal to unity. Therefore, the constant is roughly dependent on the ratio of the electron occupation numbers, which is about 1 for *σ*(1t_1_)/*σ*(4t_2_) and 0.67 for *σ*(1e)/*σ*(4t_2_) as is the case shown in Fig. 3a and b. We also illustrate in the figures the theoretical ratios for *σ*(1t_1_)/*σ*(4t_2_) and *σ*(1e)/*σ*(4t_2_) of CF_4_ calculated at the equilibrium interatomic F-F distance *R*_FF_ = 2.1551 Å^44^ as well as at the distances changing −0.2 Å, −0.1 Å,+0.1 Å,+0.2 Å. The theoretical momentum profiles are calculated by B3LYP density functional method adopting aug-cc-pVTZ basis sets (see Supplementary Information Note 2). A very sensitive dependence of the oscillation interference pattern on the interatomic F-F distance can be observed. The theoretical results at equilibrium geometry give the best agreement with the experiments.

For CO_2_ molecule, 3*σ*_*u*_ and 4*σ*_*g*_ MOs, which are hybrid orbitals of the oxygen (O) lone-pairs, are anti-symmetrical (*u*) and symmetrical (*g*) that are expected to give oscillations in antiphase. The experimental and theoretical momentum profile ratios of 3*σ*_*u*_ and 4*σ*_*g*_ MOs are shown in Fig. 3c. As is expected, the experimental ratio presents regular oscillation around a constant of about 0.85 that corresponds to the pole strength ratio of 4*σ*_*g*_ and 3*σ*_*u*_ (0.72/0.85)^45^. Similar to the situation of CF_4_, a very sensitive dependence of the interference pattern on the interatomic O-O distance is observed and the theoretical result at equilibrium geometry (*R*_OO_ = 2.3267 Å^44^) give approximately the best agreement with the experiment.

It should also be noted that the experimental data obviously deviate from the theoretical predictions at large momentum. These derivations should be ascribed to the distorted wave effect which is a common phenomena in EMS^14^ at large momentum region and such effect may be different for different MOs. However, it still remains an unresolved problem to include the distorted wave effect in the calculations for the molecular system.

### Determining interatomic distance

**Figure 4:**
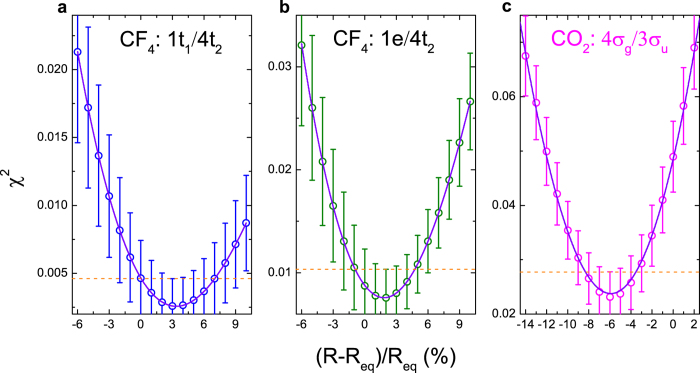
The *χ*^2^ value distributions for CF_4_ and CO_2_ as a function of interatomic distances. (**a**) for 1t_1_/4t_2_ of CF_4_, (**b**) for 1e/4t_2_ of CF_4_ and (**c**) for 4*σ*_*g*_/3*σ*_*u*_ of CO_2_.

As is discussed above, the oscillation period of the interference pattern is very sensitive to the change of interatomic distance, which provides a possible way to determine the interatomic distances with high precision. This is the well-known benefit in precision of any interferometric measurements like the Young’s double-slit experiment. In order to determine the exact values of the equilibrium interatomic distances from the present experimental data, a series of theoretical momentum profile ratios are calculated at various interatomic distances *R* and a least-square fitting procedure is performed (see Supplementary Information 4). The *χ*^2^ values, which is defined as the sum of the squared differences between experimental and theoretical momentum profile ratios, are shown as open circles in Fig. 4 as functions of relative interatomic distances (*R* − *R*_eq_)/*R*_eq_, where *R*_eq_ are the equilibrium interatomic distances of CF_4_ and CO_2_ reported in ref. 44. Three-order polynomials (solid line) are used to fit the *χ*^2^ distributions. As can be seen in Fig. 4a–c, the minimum points of *χ*^2^ values are (*R* − *R*_eq_)/*R*_eq_ = 0.033, 0.018 and −0.059 for the momentum profile ratios of 1t_1_/4t_2_, 1e/2t_2_ of CF_4_ and 4*σ*_*g*_/3*σ*_*u*_ of CO_2_. Therefore the exact values of the equilibrium interatomic distances of the present work can thus be determined to be *R*_FF_ = 2.23 Å or 2.19 Å (2.21 Å on average) for CF_4_ and *R*_OO_ = 2.19 Å for CO_2_. On the other hand, the uncertainty of *χ*^2^ value, shown as error bar in Fig. 4, can be deduced from that of the experimental data, which includes the statistical and deconvolution uncertainties. The corresponding error bars show that the minimum points of *χ*^2^ distributions can just be resolved from the points of (*R* − *R*_eq_)/*R*_eq_ = 0.00, 0.07 for 1t_1_/4t_2_ of CF_4_, (*R* − *R*_eq_)/*R*_eq_ = −0.01, 0.05 for 1e/4t_2_ of CF_4_ and (*R* − *R*_eq_)/*R*_eq_ = −0.09, −0.03 for 4*σ*_*g*_/3*σ*_*u*_ of CO_2_, as indicated by the dashed lines in Fig. 4a–c. The uncertainties of the determined values of equilibrium interatomic distances are thereby ±0.08 Å or ±0.06 Å (±0.07 Å on average) for CF_4_ and ±0.07 Å for CO_2_, which are about 3–4% of interatomic distances. By further improving the momentum resolution and reducing the statistical uncertainty, it would not be difficult to reach 1% or better in geometry determination.

## Discussion

We demonstrate a robust method for the retrieval of the interatomic distances from the multicenter interference effect of molecules with EMS. A sensitive dependence of the oscillation period on the interatomic distance is observed in the ratios of electron momentum profiles between two MOs with oscillations in antiphase. A least-square fitting procedure is used to precisely determine the equilibrium F-F distance in CF_4_ and O-O distance in CO_2_ with sub-Ångström precision. The result for F-F distance is *R*_FF_ = 2.21 Å ±0.07 Å, which is consistent with the value reported by electron diffraction^44^ within the experimental uncertainty. As for O-O distance in CO_2_, the result is determined to be *R*_OO_ = 2.19 Å ±0.07 Å. It is slightly smaller than the value from the electron diffraction experiments^44^. EMS is readily a well-established technique to obtain the spherically averaged electron momentum distributions for individual MOs. Therefore, by unveiling its new ability of determination of molecular bond lengths, EMS is now able to obtain the electron density distributions of MOs and the molecular geometry information simultaneously in one set of measurements. On the other hand, the recent advances in ultrashort electron pulses allowing one to achieve 4D electron diffraction^3^,^4^,^5^,^6^ as well as 4D electron microscopy^46^,^47^. The most recent work^48^,^49^ also demonstrated the feasibility of time-resolved EMS measurements of short-lived transient species, where an ultrashort photon pulse is used for exciting the dynamics of interest and an ultrashort electron pulse is applied to probe the system as a function of the delay time between them. Therefore, by employing the new approach of the present work as well as ultrashort electron pulses, EMS has the potential to apply to ultrafast imaging of the molecular dynamics by exploring not only the change of electron densities but also the change of molecular structures for transient species.

## Methods

### Experiment

The experiment is carried out using a high-sensitivity angle and energy dispersive multichannel electron momentum spectrometer with nearly 2*π* azimuthal angle range (2*π*-EMS). The details of the 2*π*-EMS can be seen in ref. 42. Briefly, the experiment involves coincidence detection of two outgoing electrons produced by electron impact ionization of the target molecule. The electron beam generated from a thermal cathode electron gun is accelerated to the energy of 1200 eV plus the binding energy to collide with the gas-phase target in the gas cell. The symmetric non-coplanar kinematics is employed. The scattered and ejected electrons with equal polar angles (*θ*_1_ = *θ*_2_ = 45°) and energies are analyzed by a spherical electrostatic analyzer with 90° sector and 2*π* azimuthal angle range. The two outgoing electrons are detected in coincidence by a position sensitive detector placed at the exit plane of the analyzer. The passing energies of energy analyzer are 600 eV for CF_4_ and 200 eV for CO_2_, respectively. The performances of EMS-2*π* are calibrated by electron impact ionization of Argon before experiment. The energy resolution, polar angle resolution and azimuthal angle resolution are determined to be Δ*E* = 2.2 eV, Δ*θ* = 1.0° and Δ*ϕ* = 2.4° for CF_4_ experiment and Δ*E* = 1.4 eV, Δ*θ* = 1.0° and Δ*ϕ* = 2.9° for CO_2_ experiment, respectively.

### Interference effect in EMS

Based on the LCAO approximation, the momentum space wavefunction of the *i*th MO can be expressed as,


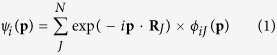


where *ϕ*_*iJ*_(***p***) is the momentum space representation of the atomic basis function on *J*th atom and ***R***_*J*_ is the coordinate vector. *N* is the number of atoms. The triple differential cross section (TDCS) of EMS is proportional to the spherically averaged electron momentum distribution (see Supplementary Information Note 2, Note3 for details) that can be separated into two parts^17^,


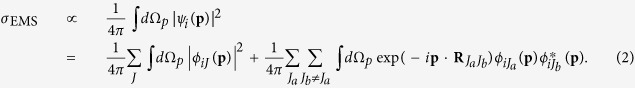


Similar to the cross section in the electron diffraction^50^ (including the laser induced electron diffraction^10^,^11^), the first term of the right side of eq. (2) is the electron density distributions contributed from the atoms, which is the incoherent sum of electron densities on individual atoms and carries no molecular structure information. While the second term of the right side of eq. (2) contains the interference factor, the oscillation periodic of which depends on the interatomic distance 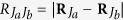 between atom *J*_*b*_ and *J*_*a*_ and the phase of which depends on the overlap of the wavefunction between different atoms. The second term can be expanded by spherical Bessel functions and the momentum profile ratio of two MOs with antiphase character can be approximately expressed as (see Supplementary Information Note 3),


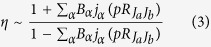


where *j*_*α*_ is the *α* order spherical Bessel function. The ratio eliminates the influence of the rapidly decreasing atomic momentum distribution, making it possible to direct observe of the interference pattern. Meanwhile, the ratio also significantly magnifies the magnitude of the interference oscillations.

## Additional Information

**How to cite this article**: Wang, E. *et al*. Imaging molecular geometry with electron momentum spectroscopy. *Sci. Rep.* **6**, 39351; doi: 10.1038/srep39351 (2016).

**Publisher's note:** Springer Nature remains neutral with regard to jurisdictional claims in published maps and institutional affiliations.

## Supplementary Material

Supplementary Information
