# Supplementary material for: Imaging molecular geometry with electron momentum spectroscopy
Source: Sci Rep. 2016 Dec 22;6:39351. doi: 10.1038/srep39351 (PMC5177885; doi:10.1038/srep39351)
Supplement: Supplementary Information [file srep39351-s1.pdf]

Supplementary Information

Imaging molecular geometry with electron momentum spectroscopy

Enliang Wang, Xu Shan, Qiguo Tian, Jing Yang, Maomao Gong, Yaguo Tang, Shanshan Niu, and Xiangjun Chen

Supplementary Figure

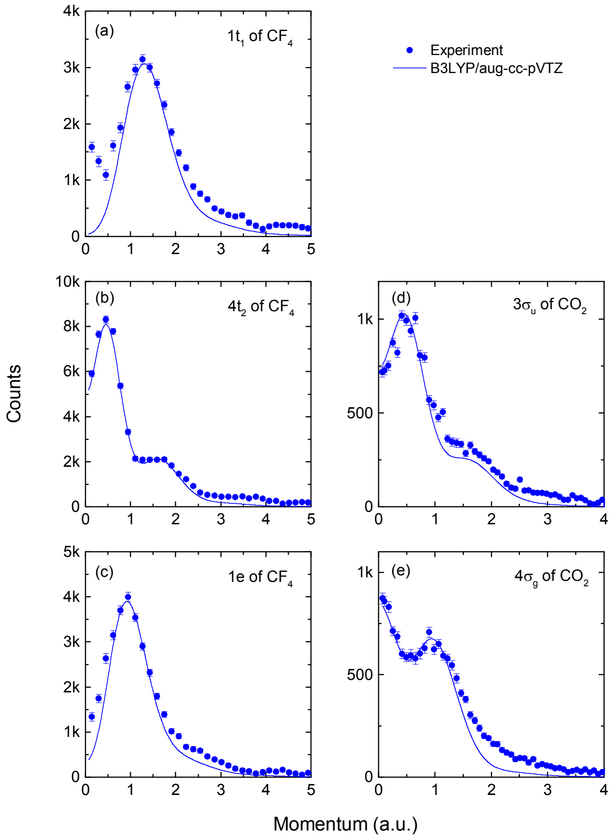

Fig. S1. The experimental and theoretical momentum profiles of the relevant molecular orbitals of CF_4 and CO_2.

Note 1: The principle of electron momentum spectroscopy

Electron momentum spectroscopy (EMS) is based on the electron-impact ionization experiment near the Bethe ridge, in which an orbital electron of target molecule is cleanly knocked out by a high-energy incident electron, and the residual ion acts as a spectator. From energy and momentum conservation, the binding energy ε_f and the momentum $\boldsymbol{p}$ of the target electron are given by,

$$\varepsilon_f = E_0 - E_1 - E_2, \tag{S1-1}$$

$$p = p_1 + p_2 - p_0, \tag{S1-2}$$

where E_i, $\boldsymbol{p}_i$ (i = 0, 1, 2) are kinetic energies and momenta of incident and two outgoing electrons, respectively. Under symmetric non-coplanar kinematics adopted in this work ($E_1 = E_2$, $\theta_1 = \theta_2 = \theta$ = 45°), the magnitude of momentum can be expressed as

$$p = \left\{ (2p_1 \cos\theta - p_0)^2 + \left[2p_1 \sin\theta \sin\left(\frac{\phi}{2}\right) \right]^2 \right\}^{1/2}, \tag{S1-3}$$

where θ is the polar angle and ϕ is the relative azimuthal angle between two outgoing electrons. Therefore, by detecting the two outgoing electrons in coincidence, the binding energy and the momentum of the target electron can be determined. Before the experiments on CF_4 and CO_2, the energy resolutions of the present EMS spectrometer are determined to be ~ 2.2 eV for CF_4 and ~1.4 eV for CO_2 (full width at half maximum) by measuring Ar 3p ionization.

On the theoretical side, within binary encounter approximation and plane wave impulse approximation, the triple differential cross section of EMS is directly proportional to the spherically averaged square of the electronic overlap[1-4] for randomly oriented molecules,

$$\frac{d^3\sigma}{d\Omega_1 d\Omega_2 dE_2} = (2\pi)^2 \frac{p_1 p_2}{p_0} f_{ee} \frac{1}{4\pi} \int d\Omega_p \left| \langle f | a(\boldsymbol{p}) | 0 \rangle \right|^2, \tag{S1-4}$$

where $|0\rangle$ is the ground state of target, $|f\rangle$ is the final ion state, $a(\boldsymbol{p})$ is the operator annihilating an electron of momentum $\boldsymbol{p}$. f_{ee} is the electron-electron collision factor. Within the weak coupling approximation, the final ion state can be expanded by orthonormal basis states $|j\rangle$ that are the linear combination of configurations formed by annihilating one electron in a target eigenstate. Then the overlap is[1, 2]

$$\langle f | a(\boldsymbol{p}) | 0 \rangle = \sum_j \langle f | j \rangle \langle j | a(\boldsymbol{p}) | 0 \rangle, \tag{S1-5}$$

where $\langle j | a(\boldsymbol{p}) | 0 \rangle$ equals to zero if $|j\rangle$ is not a single-hole states $|i\rangle$ formed by annihilating an electron from the orbital $|i\rangle$ in the target ground state. Thus Eq. (S1-5) can be deduced to

$$\langle f | a(\boldsymbol{p}) | 0 \rangle = \langle f | i \rangle \varphi_i(\boldsymbol{p}), \tag{S1-6}$$

where $\varphi_i(\boldsymbol{p})$ is known as Dyson orbital in momentum space. The EMS cross section is thus given by[1, 2]

$$\frac{d^3\sigma}{d\Omega_1 d\Omega_2 dE_2} \propto S_f^i \frac{1}{4\pi} \int d\Omega_p \left|\varphi_i(\boldsymbol{p})\right|^2, \tag{S1-7}$$

where $S_f^i = \left|\langle f | i \rangle\right|^2$ is the spectroscopic factor or pole strength which is the probability of finding a one-hole configuration in final ion state.

By further applying the target Hartree-Fock (HF) or Kohn-Shan (KS) approximation, the EMS cross section can be simplified to[5]

$$\frac{d^3\sigma}{d\Omega_1 d\Omega_2 dE_2} \propto S_f^i \frac{1}{4\pi} \int d\Omega_p \left|\psi_i(\boldsymbol{p})\right|^2, \tag{S1-8}$$

where $\psi_i(\boldsymbol{p})$ is the momentum space representation of the neutral state canonical HF or KS one-electron wavefunction of the *i*th MO from which the electron is ejected. The integral in eq. (S1-8) is known as the spherically averaged one-electron momentum distribution or electron momentum profile.

Note 2: Experimental and theoretical momentum profiles

Carbon tetrafluoride (CF_4) belongs to T_d point group symmetry. Its ground state electronic configurations based on the HF calculation is:

$$\underbrace{(1a_1)^2(2a_1)^2(1t_2)^6}_{\text{core}}\underbrace{(3a_1)^2(2t_2)^6}_{\text{inner-valence}}\underbrace{(4a_1)^2(3t_2)^6(1e)^4(4t_2)^6(1t_1)^6}_{\text{outer-valence}}$$

Carbon dioxide (CO_2) belongs to $D_{\infty h}$ point group symmetry and its ground state electronic configuration in the HF approximation is:

$$\underbrace{(1\sigma_g)^2(1\sigma_u)^2(2\sigma_g)^2}_{\text{core}}\underbrace{(3\sigma_g)^2(2\sigma_u)^2}_{\text{inner-valence}}\underbrace{(4\sigma_g)^2(3\sigma_u)^2(1\pi_u)^4(1\pi_g)^4}_{\text{outer-valence}}$$

In the present work, the two dimensional electron density maps as functions of binding energy and relative azimuthal angle for CF_4 and CO_2 are measured while the total binding energy spectra (BES) are obtained by summing over all the measured relative azimuthal angles (ϕ). A series of Gaussian functions are employed to fit the BES corresponding to the ionization from the five MOs for CF_4 and four MOs for CO_2. The peak positions of the Gaussian functions are referred to the results of high-resolution photoelectron spectra (PES) studies[6, 7]. The widths of the Gaussian peaks are the

convolution of the EMS instrumental energy resolution and the Franck-Condon widths of the ionization bands observed in the PES.

The experimental momentum profiles (XMPs) are obtained by deconvoluting the corresponding ionization peaks from the BES at different azimuthal angles ϕ and plotting the area under the fitted Gaussian peaks as a function of momentum p.

The theoretical momentum profiles (TMPs), on the other hand, are calculated according to eq. (S1-8). The corresponding position-space wavefunctions are obtained by density functional theory employed B3LYP hybrid functional[8-11] with aug-cc-pVTZ basis sets using GAUSSIAN 03 package of program[12].

For the sake of comparison with the experiments, the theoretical results are folded with the angular resolution ($\Delta\theta = 1.0^{\circ}$, $\Delta\phi = 2.4^{\circ}$ for CF_4 and $\Delta\theta = 1.0^{\circ}$, $\Delta\phi = 2.9^{\circ}$ for CO_2 determined by measuring angular distribution of Argon 3p ionization) using the Gaussian weighted planar grid method[13]. The XMPs and the TMPs are placed on a common intensity scale by normalizing the TMP of $4t_2$ MO to the corresponding XMP for CF_4 and the TMP of $4\sigma_g$ MO to the corresponding XMP for CO_2.

The XMPs and the TMPs are shown in Supplementary Figure 1, where the left column is for CF_4 and the right column for CO_2. One can see from the figures that good agreements between the XMPs and the TMPs have been achieved except in small momentum region for $1t_1$ and 1e MOs of CF_4 and in large momentum for all of the involved MOs. The derivations of TMPs from XMPs should be ascribed to the distorted wave effect[14]. Such effect can be eliminated to some extent in the cross section ratios of two MOs.

Note 3: Interference effect on electron momentum profile

Based on the linear combination of atomic orbitals (LCAO) approximation, the position space (r-space) wavefunction of ith molecular orbital (MO) can be expressed as,

$$\psi_i(\boldsymbol{r}) = \sum_{J}^{N} \sum_{j}^{n_J} c_{iJj} \phi_{Jj}(\boldsymbol{r}_J), \tag{S3-1}$$

where C_{iJj} is the expansion coefficient for jth basis function $\phi_{Jj}(\boldsymbol{r}_J)$ on Jth atom in coordinate $\boldsymbol{r}_J$ referred to the atom center. n_J and N is the number of the basis function and atoms, respectively. By Fourier transform of eq. (S3-1), the momentum space (p-space) wavefunction can be obtained,

$$\psi_i(\boldsymbol{p}) = (2\pi)^{-3/2} \sum_J^N \sum_j^{n_J} c_{iJj} \int d\boldsymbol{r} \exp(-i\boldsymbol{p}\cdot\boldsymbol{r}) \times \phi_{Jj}(\boldsymbol{r}_J), \tag{S3-2}$$

where $\boldsymbol{r} = \boldsymbol{R}_J + \boldsymbol{r}_J$ in which $\boldsymbol{R}_J$ is the coordinate vector of atom J.

Considering Born-Oppenheimer approximation, $dr \approx dr_J$, eq. (S3-2) can be simplified as[15]

$$\psi_i(\boldsymbol{p}) = \sum_J^N \exp(-i\boldsymbol{p}\cdot\boldsymbol{R}_J) \times \phi_{iJ}(\boldsymbol{p}), \tag{S3-3}$$

where

$$\phi_{iJ}(\boldsymbol{p}) = \sum_j^{n_J} c_{iJj} (2\pi)^{-3/2} \int d\boldsymbol{r}_J \exp(-i\boldsymbol{p}\cdot\boldsymbol{r}_J) \phi_{Jj}(\boldsymbol{r}_J). \tag{S3-4}$$

Eq. (S3-4) is the p-space representation of the atomic basis function $\sum_j^{n_J} c_{iJj}\phi_{Jj}(\boldsymbol{r}_J)$. Obviously, the p-space MO is also a linear combination of p-space AOs, and each term has a phase factor which preserves the geometry information.

The electron momentum profile of ith MO is defined as

$$\rho_{iT}(p) = \frac{1}{4\pi} \int d\Omega_p \left|\psi_i(\boldsymbol{p})\right|^2. \tag{S3-5}$$

By substituting eq. (S3-3) into eq. (S3-5), we get

$$\rho_{iT}(p) = \rho_{iA}(p) + \rho_{iM}(p), \tag{S3-6}$$

in which

$$\rho_{i\mathrm{A}}(p) = \frac{1}{4\pi} \sum_J \int d\Omega_p \left|\phi_{iJ}(\boldsymbol{p})\right|^2, \tag{S3-7}$$

$$\rho_{iM}(p) = \frac{1}{4\pi} \sum_J \sum_{J'\neq J} \int d\Omega_p \exp(-ip\cdot R_{JJ'}) \phi_{iJ}(p) \phi^*_{iJ'}(p), \tag{S3-8}$$

where, the $\rho_{iA}(p)$ is the electron density distributions contributed from the atoms, which is the incoherent sum of electron densities on individual atoms and carries no molecular structure information. While $\rho_{iM}(p)$ contains the interference factor depending on the interatomic distance $R_{JJ'} = R_J - R_{J'}$ between atom J' and J and the overlap of the electronic wavefunction between

atoms.

Writing the basis function $\phi_{Jj}(\mathbf{r}_J)$ as product of radial part R_{nl} and angular part Y_{lm}

$$\phi_{Jj}(r_J) = N_{nlm} R_{nl}(r_J) Y_{lm}(\Omega_{r_J}), \tag{S3-9}$$

and using the identity

$$\exp(-ip \cdot r_J) = 4\pi \sum_{lm} (-i)^l j_l(pr_J) Y_{lm}(\Omega_p) Y^*_{lm}(\Omega_{r_J}), \tag{S3-10}$$

the corresponding p-space basis function of eq. (S3-4) can be expressed as

$$\phi_{iJ}(p) = \sum_j^{n_J} c_{iJj} N_{nlm} P_{nl}(p) Y_{lm}(\Omega_p), \tag{S3-11}$$

where $P_{nl}(p) = (2/\pi)^{1/2}(-i)^l \int r_J^2 dr_J j_l(pr_J) R_{nl}(r_J)$ is the radial part, in which j_l is the l order spherical Bessel function.

Substituting eq. (S3-11) into (S3-8), we get[15]

$$\rho_{iM}(p) = \sum_{J_a} \sum_{J_b \neq J_a} \sum_{j_a} \sum_{j_b} C_{iJj} C_{iJ'j'} N_{n_a l_a m_a} N_{n_b l_b m_b} P_{n_a l_a}(p) P^*_{n_b l_b}(p)$$
$$\times \sum_{lm} (-i)^l Y^*_{lm}(\Omega_{R_{J_a J_b}}) (-1)^{m+m_a+m_b} \sqrt{\frac{(2l+1)(2l_a+1)(2l_b+1)}{4\pi}} \begin{pmatrix} l & l_a & l_b \\ -m & -m_a & -m_b \end{pmatrix} \begin{pmatrix} l & l_a & l_b \\ 0 & 0 & 0 \end{pmatrix} j_l(pR_{J_a J_b}) \cdot \tag{S3-12}$$

For the MOs in complete antiphase, e.g. $\psi_i = C_1\phi_1 + C_2\phi_2$ and $\psi_j = C_1\phi_1 - C_2\phi_2$, the momentum profile ratio of the two MOs can be expressed as

$$\frac{\rho_{iT}(p)}{\rho_{jT}(p)} = \frac{\rho_A(p) + \sum_\alpha A_\alpha j_\alpha(pR_{J_a J_b})}{\rho_A(p) - \sum_\alpha A_\alpha j_\alpha(pR_{J_a J_b})}. \tag{S3-13}$$

Usually, $\rho_A(p)$ and $P_{n_a l_a}(p) P^*_{n_b l_b}(p)$ are slowly varying functions compared to the spherical Bessel function and can be regarded as constants. Eq. (S3-13) can be simplified as

$$\eta = \frac{\rho_{iT}(p)}{\rho_{jT}(p)} \sim \frac{1 + \sum_\alpha B_\alpha j_\alpha(pR_{J_a J_b})}{1 - \sum_\alpha B_\alpha j_\alpha(pR_{J_a J_b})}. \tag{S3-14}$$

In this way, the influence of the rapidly decreasing atomic momentum distribution is eliminates and the magnitude of the interference oscillation is significant magnified.

Note 3: Least-square fitting procedure

The function of the least-square fitting is defined as

$$\chi^2(R,\alpha) = \frac{1}{N}\sum_i^N \left[\alpha\eta_{\text{the}}(R,p_i) - \eta_{\exp}(p_i)\right]^2 . \quad \text{(S4-1)}$$

where η_{the} and $\eta_{\exp}$ are theoretical and experimental momentum profile ratios and i refers to the discretized points of the momentum p. R is the interatomic distance and α is the scaling factor of the theoretical momentum profile ratio. The interatomic distance is determined as follows: first, the χ^2 value is calculated at various relative interatomic distances, $(R-R_{eq})/R_{eq}$ = -6% - 10% for CF_4 and $(R-R_{eq})/R_{eq}$ = -14% ~ 2% for CO_2 with length step of 1%, where R_{eq} is the equilibrium interatomic distances reported in ref. 16 (R_{eq}=2.1551 Å for CF_4 and R_{eq}= 2.3267 Å for CO_2). Parameter α is adjusted to obtain minimum χ^2 value at each interatomic distance. Then a least-square fitting procedure is carried out to determine the global minimum point of χ^2 distribution and thereby the equilibrium interatomic distance of the present work is determined.

The uncertainty of χ^2 value can be deduced from the experimental errors

$$\delta(\chi^2(R,\alpha)) = \frac{2}{N}\sqrt{\sum_i^N \left[\alpha\eta_{\text{the}}(R,p_i) - \eta_{\exp}(p_i)\right]^2 \delta^2(\eta_{\exp}(p_i))} \quad \text{(S4-2)}$$

The uncertainty of the equilibrium interatomic distance can be estimated from the χ^2 distribution and its errors.

1. McCarthy I. E. & Weigold E. Electron momentum spectroscopy of atoms and molecules. *Reports on Progress in Physics* **54**, 789 (1991).
2. Weigold E. & MaCarthy I.E. *Electron Momentum Spectroscopy*. Kluwer Academic/Plenum Press (1999).
3. Coplan M. A., Moore J. H. & Doering J. P. (e, 2e) spectroscopy. *Rev. Mod. Phys.* **66**, 985-1014 (1994).
4. Brion C. E. Looking at orbitals in the laboratory: The experimental investigation of molecular wavefunctions and binding energies by electron momentum spectroscopy. *International Journal of Quantum Chemistry* **29**, 1397-1428 (1986).
5. Duffy P., Chong D. P., Casida M. E. & Salahub D. R. Assessment of Kohn-Sham density-functional orbitals as approximate Dyson orbitals for the calculation of electron-momentum-spectroscopy scattering cross sections. *Phys. Rev. A* **50**, 4707-4728 (1994).
6. Carlson T. A., *et al*. Angle-resolved photoelectron cross section of CF_4. *J. Chem. Phys.* **81**, 3828-3834 (1984).
7. Kimura K., Katsumata S., Achiba Y., Yamazki T. & Iwata S. *Handbook of HeI Photoelectron Spectra of Fundamental Organic Molecules : Ionization Energies, ab initio Assignments, and Valence Electronic Structure for 200 Molecules*. Halsted Press (1981).
8. Duffy P., Chong D. P., Casida M. E. & Salahub D. R. Assessment of Kohn-Sham density-functional orbitals as approximate Dyson orbitals for the calculation of electron-momentum-spectroscopy scattering cross-sections. *Phys. Rev. A* **50**, 4707-4728 (1994).

9. Casida M. E. Generalization of the optimized-effective-potential model to include electron correlation - a variational derivation of the Sham-Schluter equation for the exact exchange-correlation potential. *Phys. Rev. A* **51**, 2005-2013 (1995).
10. Lee C. T., Yang W. T. & Parr R. G. Development of the Colle-Salvetti correlation-energy formula into a functional of the electron-density. *Phys. Rev. B* **37**, 785-789 (1988).
11. Becke A. D. Density-functional thermochemistry. III. the role of exact exchange. *J. Chem. Phys.* **98**, 5648-5652 (1993).
12. Frisch M. J., *et al*. GAUSSIAN 03, Revision B. 04, Gaussian, Inc, Pittsburgh, PA, 2003.
13. Duffy P., Cassida M. E., Brion C. E. & Chong D. P. Assessment of Gaussian-weighted angular resolution functions in the comparison of quantum-mechanically calculated electron momentum distributions with experiment. *Chem. Phys.* **159**, 347-363 (1992).
14. Ren X. G., *et al*. Direct observation of distorted wave effects in ethylene using the (e, 2e) reaction. *Phys. Rev. Lett.* 94, 163201 (2005).
15. Cook J. P. D. & Brion C. E. Binary (e, 2e) spectroscopy and momentum-space chemistry of CO_2. *Chem. Phys.* **69**, 339-356 (1982).
16. Fink M., Schmiedekamp C. W. & Gregory D. Precise determination of differential electron scattering cross sections. II. CH_4, CO_2, CF_4. *J. Chem. Phys.* **71**, 5238-5242 (1979).
